# Comparison of American Quarter Horses Competing in Western Pleasure, Hunter under Saddle, and Reining Using Linear Traits

**DOI:** 10.3390/ani11102861

**Published:** 2021-09-30

**Authors:** Isabel Thea Roth, Britta Schielke, Markus Rensing, Maren Bernau

**Affiliations:** 1 Fakultät Agrarwirtschaft, Volkswirtschaft und Management, Hochschule für Wirtschaft und Umwelt Nürtingen-Geislingen, Neckarsteige 6-10, 72622 Nürtingen, Germany; maren.bernau@hfwu.de; 2 Independent Researcher (former: Deutsche Quarter Horse Association Breeding Management), Wickengartenstraße 3, 35428 Langgöns, Germany; britta@ing-schielke.de; 3 Independent Researcher (former: Deutsche Quarter Horse Association Breeding Management), Astenstraße 13, 57392 Schmallenberg, Germany; markus.rensing@t-online.de

**Keywords:** American quarter horse, western pleasure, hunter under saddle, reining, linear traits

## Abstract

**Simple Summary:**

This study analyzed differences between successful American Quarter Horses competing in different disciplines by using linear traits. The goal was to detect whether American Quarter Horses successful in specific disciplines significantly differed in exterior traits. Traits were measured analogically and photometrically. Differences in lengths, angles, ratios, and circumferences were detected. Further studies are needed to better examine the specialized traits and the effect of genomic variance.

**Abstract:**

To investigate differences in American Quarter Horses conformation suggesting specialization and subpopulations within the breed, a total of 45 horses were studied. These horses were classified according to their specific sport discipline: Western Pleasure (WPL, *n* = 15), Hunter under Saddle (HUS, *n* = 15), and Reining (RN, *n* = 15). Fifteen linear traits (comprising lengths, angles, and circumferences) were measured analogically and photometrically. Afterwards, 9 ratios and differences were calculated, so that a total of 24 traits were examined. The results showed significant differences between horses depending on their sport discipline. HUS horses were significant taller and were characterized by higher values in (nearly) all length traits; they were followed by WPL and RN horses. RN horses displayed the lowest values in length traits but the largest difference between height at withers and height at croup. Nine parameters were analyzed through correlations to the height at withers; all differences were significant, with high to moderate correlation coefficients. The detected differences between the groups support recent studies on the conformation and genotype of subpopulations within a breed and reveal new findings in relation to the selected disciplines. Image analysis worked well and provided reliable data; therefore, this method can be used to examine horses in a time-efficient manner, reducing the stress caused to the animal. Further studies are required to gain more information and to associate the features of AQH anatomic structures with successful performance.

## 1. Introduction

American Quarter Horses (AQH) were originally bred as workhorses, specifically, for transportation and agriculture but became globally popular for their use in sports and leisure, resulting in approximately 2.78 million registered individuals [1]. A broad variety of modern disciplines offers opportunity for specialization, differently from other breeds, due to their increasing differentiation based on performance characteristics (i.e., agility or obedience). This has been leading to a subdivision of the breed in different lines [2,3,4], causing a specialization in breeding and the determination of measurable genomic differences between different groups according to the performed sport discipline [2,3,4,5,6], implying the existence of subpopulations within the breed. Racing and Cutting horses have been compared [2,5,6] as well as Racing, Cutting, Western Pleasure, Reining, Working Cow, and Halter horses [3,4]. These studies mainly compared genetic features, but phenotypic data are relevant as well.

In other disciplines, it is already well known that different traits of conformation may promote the use of horses in specific disciplines and are also related to a horse health status [7,8,9,10,11]. Differences in conformation between horses bred for a specific use within breeds are measurable [2,12,13,14]. Morphological traits can also show measurable differences between horses of different generations whose breeding was focused on performance in sports such as dressage [14]. For breeding purposes, measurable traits have higher heritability than judged conformation traits [15,16,17]. Measurements can be based on photometrical measuring [18], measures taken directly on the animal [10,18,19,20], or measurements in motion using markers [21]. To our knowledge, only a few studies exist about linear traits in AQH in relation to sports performance, especially with respect to these three disciplines. For example, the study by Meira et al. [3] revealed significant differences between cutting and racing horses in all physical traits. 

Therefore, the aim of the present study was to evaluate if measurable differences in linear traits of conformation can be found between horses involved in the disciplines Western Pleasure (WPL), Hunter under Saddle (HUS), and Reining (RN), indicating an indirect selection for traits not necessarily considered for scoring or breeding goals, as described by Holmström et al. [7]. Although AQH are required to meet a breeding goal including a specific height at the withers (145 to 165 cm) and defined morphologic traits [2], the various disciplines differ in their specific performance needs [22]: in WPL and HUS, emphasis lies on quality and correctness of movement and gaits, while RN horses should be able to perform specific maneuvers such as spins and sliding stops with athletic ability and obedience [22]. Therefore, WPL and HUS horses have been merged into one group in recent studies of AQH subpopulations. WPL horses should show free-flowing strides of reasonable length in relation to their conformation, covering a reasonable amount of ground with little effort and being a pleasure to ride [18], usually showing very slow and gathered movements. HUS horses should show free-flowing, efficient ground covering, and athletic movement with long, low strides reaching forward with ease and smoothness [22], usually resulting in higher speed compared to WPL horses, since stride length and speed are positively correlated [23]. RN horses are primarily judged on the execution of certain maneuvers such as sliding stops, spins, and circles with different tempi, requiring maneuverability and acceleration [24].

These different requirements suggest that morphological differences between horses used in these three disciplines are measurable. To detect differences and evidence of further specialization in AQH, linear traits such as length, angles, and circumference of different body parts were measured in the present study, focusing on AQH performing successfully in one of the mentioned disciplines.

## 2. Materials and Methods

### 2.1. Animals 

In total, 45 AQH, registered in the Deutsche Quarter Horse Association (DQHA), a German affiliate to the American Quarter Horse Association (AQHA), were studied. These 45 horses included 15 horses per discipline (WPL *n* = 15; HUS *n* = 15; RN *n* = 15). All horses were at least four years old, and their sex was not considered. This was due to the fact that the horse sex is not considered in judging of the described disciplines. Horses above four years can compete against horses of every other age [22]. 

Competing horses are judged independently of their sex in all disciplined described in the present study. Stallions, geldings, and mares compete together and are judged according to the same regulations. Our aim was to identify if there might be differences in conformation between horses of the same breed performing in those disciplines. Therefore, the aim was to stay as close to the practice of the competitive judging situation as possible, basing the selection of horses purely on sports performance [22]. 

Therefore, the premise for entering this study was a successful performance at the International German Championship 2019 in one of the examined disciplines (WPL, HUS or RN) and a minimum age of four years. 

### 2.2. Measurements

**Figure 1:**
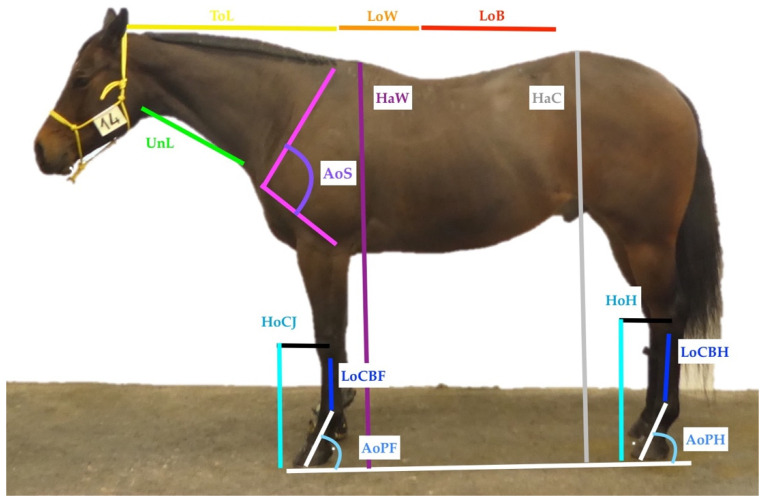
Positioning of the linear measurements. Abbreviations: Height at Withers (HaW), Height at Croup (HaC), Topline (Tol), Underline (UnL), Angle of shoulder (AoS), Length of withers (LoW), Length of back (LoB), Height of carpal joint (HoCJ), Height of hock (HoH), Length of cannon bone forelimb (LoCBF), Length of cannon bone hindlimb (LoCBH), Angle of pastern forelimb (AoPF), Angle of pastern hindlimb (AoPH).

At the International German Championship, the successful horses were photographed under similar conditions provided by an experimental setup including markings on the floor, a camera tripod, and a digital camera (Panasonic DMC-TZ81, Osaka, Japan) (see Supplementary Material Figure S1). The horses were placed parallel to the floor line (Figure 1) with similar posture considering limb position and head posture. If the horses could not be placed exactly parallel to the floor line, a line parallel to the horse’s feet was added digitally. Each horse was equipped with a continuous number on size-defined plates in order to identify them and as one of two calibration tools for digital measurements. The set-up was not changed during the data gathering process, and the zoom function of the camera was not used. The markings on the floor were 3.5 m apart and marked in sections, so that the camera could be aligned to the positioning of the horse without changing the distance between horse and camera.

Pictures were taken only from the side, and only one picture per horse was used for the measurements to maintain the same angle and distance from the camera for all measurements. Measurements of the proximal hind limb were not taken, since the training status of the horses did not allow a definite location of the bone structure due to the muscle mass.

During the championship, three variables were measured on the live animal (height at withers (HaW) and circumference of the cannon bone of the forelimb (CircCBF) and of the hindlimb (CircCBH)). Additionally, a photometrical measurement was implemented for 12 linear traits (see Table 1 and Figure 1), using the software Synedra View Personal (Synedra information technologies GmbH, Innsbruck, Austria) a DICOM (Digital Imaging and Communications in Medicine) viewer [25]. To achieve correct measurements, the scale of the software was calibrated by using the analogically measured height at withers (HaW) and was controlled by using the size-defined number plates attached to the horse’s halters (Figure 1), providing measurements accurate to one decimal place. Two factors were used to calibrate the software and verify the calibration. The HaW was measured analogically as a reference using a measuring device, giving 1 d.p. exact measurements. This 1 d.p. exact measurement and the number plate, whose size was also measured 1 d.p. exact, were used to calibrate and check the measurement software. The Synedra viewer needed one measurement for calibration, therefore, the number plate was used to calibrate the software tool. The HaW was then used to verify the calibration. The results of the analogical and photometrical measurements of HaW were compared. The results of the digital measurement had to be 1 d.p. exact compared to the analogical measurements of both the number plate and HaW before using the calibration to measure other traits. 

In total, 15 variables were measured (see Table 1). All selected variables were chosen based on breeding usability as well as performance suitability [3,22,26,27].

Further parameters were calculated to obtain insights beyond possible differences in lengths as a result of different body sizes, using the analogically and digitally measured traits (see Table 2).

### 2.3. Statistical Analysis

The 15 measured and nine calculated traits resulted in a total of 24 variables, which were generated and statistically analyzed using R-statistics (Version 3.6.3; R-Foundation, Vienna, Austria) and SPSS Statistics (IBM Corp, Armonk, NY, USA). The whole dataset was tested for normal distribution using the Shapiro–Wilk test provided by R. Normally distributed data were tested by a comparison of means using analysis of variance (ANOVA) including post-hoc testing with Bonferroni correction provided by SPSS (see Table 3). Nonnormally distributed data were tested by a comparison of means using the Kruskal–Wallis test including post-hoc-testing with Bonferroni correction provided by SPSS (see Table 3). Correlations of nine traits to the HaW were determined (see Table 4). Additionally, the frequency of the compliance of the HaW stated in the breed description (see Supplementary Material Tables S1 and S2) and the frequency of a greater HaC compared to the HaW (see Supplementary Material Tables S3 and S4) were calculated using SPSS.

## 3. Results

### 3.1. Comparison of the Different Traits by Discipline

Of these 24 variables, 15 showed significant differences when comparing horses grouped according to their discipline (see Table 3). Of these, only two calculated variables showed significant differences, all other were linear traits or circumferences.

Six variables (HaW, HaC, ToL, UnL, HoCJ, LoCBF) showed significant differences between all three groups. For these six variables, the highest values were measured for HUS horses, followed by WPL ones, and the lowest values were measured for RN horses. When comparing HaW and HaC horses, HUS horses showed similar results between both measurements (HaW: 164.0 ± 4.8 cm; HaC: 164.2 ± 5.6 cm), whereas RN horses, in particular, showed higher values for HaC (151.0 ± 2.1 cm) compared to HaW (147.8 ± 2.9 cm). HoCJ was the lowest for RN horses (42.7 ± 1.4 cm), with only slight but significant differences when compared to WPL (45.0 ± 1.7 cm) and HUS (47.5 ± 2.0 cm) horses. These results showed that HUS horses are taller than WPL and RN animals, especially regarding the length variables. RN horses had a higher HaC in comparison to HaW, whereas WPL and HUS horses showed nearly similar lengths for HaC and HaW. This was confirmed by variable Diff_HaC_HaW. 

Six variables (LoB, HoH, LoCBH, CircCBF, CircCBH, Diff_HaC_HaW) showed a significant difference when comparing HUS and RN horses, while for WPL horses, we measured intermediate values. For all these variables, with the exception of Diff_HaC_HaW, HUS horses reported higher values than RN horses. Although significant differences were measured between HUS and WPL animals regarding HaW and HaC, no differences were found regarding the circumferences at the front or the back between horses involved in these two disciplines.

For two variables (LoW and Ratio_HaW_CircCBH), a significant difference was detected between RN horses and horses involved in the other two disciplines, while WPL and HUS horses were not significantly different from each other. 

Only for AoS a significant difference between HUS and the other two groups of horses (WPL and RN) was found, with HUS horses having a significant larger angle (99.5 ± 2.5°) than WPL (96.8 ± 3.0°) and RN (96.8 ± 3.3°) ones.

No significant difference was detected for eight variables, including two angle measurements, four calculated differences, and two calculated ratios (Table 3).

### 3.2. Correlations to the Height at Withers (HaW)

Correlations to the HaW were calculated for nine parameters (LoW, LoB, Diff_HaC_HaW, LoCbF, LoCbH, AoS, HoCJ, HoH, and CircCbH). All correlations were significant (*p* < 0.05), with eight parameters positively correlating (LoW, LoB, LoCbF, LoCbH, AoS, HoCJ, HoH and CircCbH), and one (Diff_HaC_HaW) negatively correlating (Table 4). The highest correlation to HaW was found for HoCJ, followed by HoH and CircCBH. Interestingly, LoCBF showed a higher correlation to HaW than LoCBH, although HoH showed a high correlation to HaW.

A frequency analysis showed a significant result for HUS horses, showing more likely an HaW outside the range indicated for that breed (χ^2^(2) = 13.07, *p* = 0.001, V = 0.54) (see Supplementary Material Tables S1 and S2).

## 4. Discussion

Linear traits showed significant differences between horses trained in different disciplines (Table 3). Although different disciplines were compared in the present study with respect to the work of Meira et al. [3], the results confirmed previous findings of significant changes in the physical traits of AQH. Additionally, the present study underlines the relation between linear traits and function of the animal stated by Meira et al. [3]. In the present study, new findings have been achieved. We used methods that have not been applied previously in the context of AQH involved in the disciplines of WPL, HUS, and RN. This provides information on the modern AQH breed and should be discussed for further breeding purposes. 

### 4.1. Evaluation of Linear Traits

The evaluation of linear traits is time-consuming and may be stressful for the animal. Due to this issue, the present study was based on digital image analysis of photographs, taken at the International German Championship. At the competition, only three measurements were taken analogically (HaW, CircCbF, CircCbH). Since circumferences would not have been accurately computed using two-dimensional photometric measurements, they were taken analogically, as well as HaW, for verifying photometrical calibration.

Additionally, a photograph was taken. This image was evaluated afterwards, since the horses were measured at the competition between classes, and the measuring process had to be time-efficient. Photometrical measures are a usable alternative to analogical measurements, as already stated by dos Santos et al. [18] and shown in practice in other studies such as that of Anderson and McILWraith [27].

The method worked well, and linear traits could be measured easily and in a time-efficient manner. However, some limitations were obvious and should be managed in further studies, especially concerning measurements of the upper hind limb. The muscular state of the horses did not allow a visual determination of the bone structure of the knee, hip bone, or *Tuber ischiadicum* (Figure 1). This might be solved using adhesive markings attached after palpation, as they have been used in measurements directly on the horse or in motion [10,19,21]. This was not implemented in this study, in agreement with Komosa et al. [20] and Sánchez-Guerrero et al. [14], since the muscular state of the horses was causing difficulties in locating bone structures through palpation, especially on the proximal hind limb. An inclusion of the pelvis would nevertheless offer the possibility to measure more traits and be of interest, since angles in the hind limb such as hip joint and femur angle have been shown to be influential on the performance [7]. As stated by Gmel et al. [17], the side of the photograph and posture did not have an effect on shape measurement but might influence some measured traits. Therefore, additional measurements from the back and front could allow determining more traits. In addition, repeated photographs could be used for further investigations, and in complex models, age, sex, and weight could be considered as well.

Taking measurements at an international competition had advantages: the fact that phenotypes may also be influenced by feeding, training, or other factors can be neglected in the present study, as all horses included in this study were successful at the competition, and photographs and measurements were taken at the competition.

### 4.2. Linear Traits Compared with Respcet to the Discipline

Traits connected to body size such as LoCBF, HoH, HoCJ, ToL, and UnL showed significant differences when comparing the three groups of horses, as did body size (HaW and HaC, Table 3). Especially, lengths showed significant differences, in most cases with HUS horses reporting the greatest, and RN horses the smallest length. This is in accordance with the theory of horses being proportional in size [27,28]. In the present study, HUS horses had an HaW of 164.0 ± 4.8 cm, which is at the highest limit of the height range stated in the breed description (with 165 cm maximum [2]) and more often showed an HaW outside the limits of the breed description (DQHA, 2014) (Table 3; confirmed by the frequency analysis Tables S1 and S2). RN horses had an HaW of 147.8 ± 2.9 cm, which is on the lower limit of the height range for that breed [2] (Table 3). WPL horses reported intermediate values, corresponding to an average of 157.7 ± 3.5 cm (Table 3). This might indicate that specialization has taken place, and that variety and range of phenotypes have increased since the breed description was officially established. This is in accordance with Vicente et al. [13], who showed that height at withers of a breed can have both a positive and a negative effect on performance in different disciplines.

All analyzed traits showed positive correlations with HaW, except for (Diff_HaC_HaW), which correlated negatively with it (Table 4). Interestingly, RN horses had a higher HaC than HaW, whereas for WPL and HUS horses, both lengths were nearly similar. HUS horses showed a smaller difference than RN animals, which may be linked to their respective disciplines. Longer forelimbs and longer limbs, in general, might contribute to a bigger reach in stride for HUS horses in accordance to Baban et al. [12]. Longer hindlimbs might contribute to stops and spins by enabling the hind hooves to be placed under the center of gravity. This might provide more balance and a smaller turning radius. The higher difference in RN horses might also be linked to greater muscles, rather than bone structure, in the hindlimb, as required by the specific maneuvers of this discipline. A higher croup compared to HaW might influence HUS and RN horses’ success. Further studies are needed to relate functional linear traits to specific movements of the different disciplines, as demonstrated for other breeds [12,13,14].

For most lengths, WPL horses showed intermediate values in comparison to HUS and RN animals. This might be explained by the characteristics of the respective disciplines, which differ regarding movement and maneuvers [22,24], with moderate and great ground covering stride length for WPL and HUS horses, respectively, and specific maneuvers, athletic ability, and obedience for RN horses [22,24]. To perform in the best way possible in one discipline, specialization seems to have taken place. Taller horses might be favored for HUS, since longer limbs contribute to a longer stride [8,10,12,13,14,23,29], promoted in this discipline, which also may benefit from high speed. A long stride might contribute to performance in WPL as well, but longer limbs and therefore high speed are not specifically required [22]. This might result in a smaller HaW in this group compared to the HUS group. This might be confirmed by the differentiation between cutting and racing horses, leading to cutting horses being shorter and more compact, and racing horses being taller with longer legs [3]. This must be considered, as Hildebrand [29] stated that increasing the body size might result in slower motions, but if the leg length is relative greater than the chest–rump length, a compromise is found. 

RN maneuvers require maneuverability, acceleration, and a specific speed range. Increased height and, therefore, increased mass could limit acceleration and maneuverability, as would long levers such as long limbs, since especially the front legs have to move particularly fast during spins and rollbacks. While longer limbs could allow maximum speed, the negative effects seem to prevail, resulting in smaller horses. These results cannot be directly compared to the results of Meira et al. [3], as RN horses were not evaluated. 

AoS in HUS horses was significantly different (99.5 ± 2.5°) from that of the two other groups of horses (WPL = 96.8 ± 3.0°; RN = 96.8 ± 3.3°; Table 3), with the biggest angle and, therefore, the steepest shoulder. Results in other breeds might explain the resulting effects: in Swedish warmblood horses, a long, flat shoulder had a positive effect on gait scores [7,30], and in Pura Raza Española horses, flatter shoulders can contribute to maximum stride length [14]. Since warmblood horses usually show higher knee action, which is not preferred in HUS horses, a steeper shoulder might promote a flat stride. Restriction of the stride length through a steeper shoulder may be compensated through longer limbs. A steeper shoulder might not be suitable for WPL and RN horses, since WPL horses should show a big stride in relation to their body size, and therefore, a compensation through longer limbs might not contribute to performance. RN horses are not primarily judged on their gaits but on maneuvers. A steeper shoulder and, therefore, a flatter stride in RN horses might not improve success in this discipline, since the limitation of stride length might have a negative impact on ground coverage at maximum speed and reach of the forelimbs in spins and stops, resulting in slower spins and shorter, less smooth stops. 

Other angle measurements did not show significant differences (Table 3). This has to be discussed, whether there is a methodically issue or there are, actually, no detectable differences. Because bone structures were not visible and we did not use markers on the horse while photographs were taken, these angle measurements may not be reliable. This is in agreement with Anderson and McIlwraith [27], who stated that the shoulder angle should be measured using markers after palpation. This aspect should be optimized in further studies, using markers on bone structures to be able to measure angles reliably on photographs later on. Gmel et al. [17] described the influence of posture on poll and neck-shoulder blade angles, which were not considered in the present study, as posture was standardized.

The only significant difference regarding the ratios was found for Ra_HaW_CircCBH, which showed significant differences when comparing RN and HUS horses. RN horses showed a lower mean, indicating a higher circumference in relation to body size and, therefore, more stable hindlimbs. This might be an effect of an indirect selection of RN horses with more stable hindlimbs, which might increase the ability to endure stress during stops and spins. The increased body size of HUS horses might not be the result of stability selection during breeding but could be connected to an overall size increase, as suggested for Pura Raza Española horses [14]. However, since HUS and WPL horses showed no significant difference, it is more likely that RN horses have developed a greater stability. This is confirmed by Hildebrand [29], who stated that maneuverability is favored by a small body size (in the present study, RN horses had the smallest HaW and HaC), but inertia increases with mass. Therefore, the present study includes the association of increased stability with a smaller body size, which seems to be more successful to fulfil the characteristics required for the RN discipline [22].

The examined disciplines are not necessarily the only definable disciplines for which it is possible to observe differences in the exterior appearance of successfully competing AQH. Therefore, further studies are needed to extend our knowledge of different types of AQH. In addition, considering age, sex, and weight might be of interest, as sex can influence performance [7,31]. The size of the animal might have an effect when comparing different horses [17]. However, differences in phenotypic traits between HUS, WPL, and RN horses were not observed, suggesting that the breed has undergone specialization for specific use, as it has been shown for Pura Raza Española horses [10,14]. Further studies could examine genomic or phenotypic traits in relation to sport disciplines and for breeding purposes. In further studies, the number of horses as well as their geographic location should be extended. Additionally, the examined phenotypic traits should be compared to genotypic data.

## 5. Conclusions

To sum up, significant differences were detected between three selected groups of AQH (WPL, HUS, and RN). Of 24 generated linear traits, 15 showed significant differences when comparing the three groups. Only three traits were measured analogically, all other traits were evaluated by digital analysis of photographs taken at the competition. A differentiation of horse lines based on the specific disciplines, as stated by other publications, is suggested. Due to the focus on only three disciplines and the limited number of animals, further studies, including more animals and more traits and comparing phenotypic observations to genotypic data should be conducted. These studies may provide more information on the relationship between anatomic features and function, allowing the breeding of healthy and successful AQH.

## Figures and Tables

**Table 1:** Definition of the analogically and photometrically measured traits, with abbreviations.

Trait	Abbreviation	Method	Definition
Height at withers	HaW	Analogue + digital	Vertical distance between highest point of the withers and ground.
Height at croup	HaC	digital	Vertical distance between highest point of the croup and ground.
Topline	ToL	digital	Length between presumably first cervical vertebra *(Atlas)* and the visible beginning of the withers (ca. *Vertebrae thoracicae III*).
Underline	UnL	digital	Length from *Angulus mandibulae* to *Fossa jugularis.*
Angle of shoulder	AoS	digital	Angle measured between alignment of the shoulder (*Spina scapulae)* and middle of presumable *Articulatio cubiti*.
Length of withers	LoW	digital	Length between visible beginning of the withers (ca. *Vertebra thoraciae III)* and end of visible withers (ca. *Vertebrae thoracicae VIII or IX)*.
Length of back	LoB	digital	Length between end of visible withers (ca. *Vertebrae thoracicae VIII or IX*) and presumably last lumbar vertebra (*Vertebrae lumbales VI*).
Height of carpal joint	HoCJ	digital	Vertical distance between the middle of the carpal joint (*Articulatio carpi*) and ground.
Height of hock	HoH	digital	Vertical distance between the middle of the tarsal joint (*Articulatio tarsi*) and ground.
Length of cannon bone forelimb	LoCBF	digital	Length of the cannon bone *(Os metacarpale III)* in the left forelimb (from visible middle of *Articulationes carpometacarpeae* to visible middle of *Articulatio metacarpophalangea)*.
Length of cannon bone hindlimb	LoCBH	digital	Length of the cannon bone *(Os metatarsale III)* in the left hindlimb (from visible middle of *Articulationes tarsometatarseae* to visible middle of *Articulatio metatarsophalangea)*.
Circumference of the cannon bone forelimb	CircCBF	analogue	Circumference of the left forelimb at the middle of the cannon bone *(Os metacarpale III)*.
Circumference of the cannon bone hindlimb	CircCBH	analogue	Circumference of the left hindlimb at the middle of the cannon bone *(Os metatarsale III)*.
Angle of pastern forelimb	AoPF	digital	Angle between the pastern axis of the left forelimb and the horizontal.
Angle of pastern hindlimb	AoPH	digital	Angle between the pastern axis of the left hindlimb and the horizontal.

**Table 2:** Definition of the calculated differences and ratios, with abbreviations.

Trait	Abbreviation	Definition
Difference height at croup—height at withers	Diff_HaC_HaW	Difference between height at croup and height at withers.
Difference topline—underline	Diff_ToL_UnL	Difference between lengths of topline and underline.
Difference hock—carpal joint	Diff_HoH_HoCJ	Difference between height of hock and height of carpal joint.
Difference length of cannon bone hindlimb—forelimb	Diff_LoCB	Difference between length of the cannon bone of the hindlimb and length of the cannon bone of the forelimb.
Difference angle of pastern forelimb—hindlimb	Diff_AoP	Difference between the angle of the pastern of the forelimb and the angle of the pastern of the hindlimb.
Ratio length of cannon bone forelimb to height at withers	Ra_HaW_LoCBF	Ratio of the length of the cannon bone of the forelimb to the height at withers.
Ratio length of cannon bone hindlimb to height at withers	Ra_HaW_LoCBH	Ratio of the length of the cannon bone of the hindlimb to the height at withers.
Ratio height at withers to circumference of cannon bone forelimb	Ra_HaW_CircCBF	Ratio of the circumference of the cannon bone of the forelimb to the height at withers.
Ratio height at withers to circumference of cannon bone hindlimb	Ra_HaW_CircCBH	Ratio of the circumference of the cannon bone of the hindlimb to the height at withers.

**Table 3:** Comparison by means using the ANOVA model including post hoc analysis and Bonferroni correction (level of significance *p* < 0.05; means ± standard deviation).

Trait	WPL	HUS	RN
Height at withers (HaW) [cm]	157.7 ± 3.5 ^a^	164.0 ± 4.8 ^b^	147.8 ± 2.9 ^c^
Height at croup (HaC) [cm]	159.5 ± 5.2 ^a^	164.2 ± 5.6 ^b^	151.0 ± 2.1 ^c^
Topline (ToL) [cm]	82.0 ± 3.9 ^a^	86.1 ± 4.1 ^b^	74.6 ± 4.7 ^c^
Underline (UnL) [cm]	51.0 ± 4.1 ^a^	54.9 ± 4.5 ^b^	44.3 ± 3.7 ^c^
Height of carpal joint (HoCJ) [cm]	45.0 ± 1.7 ^a^	47.5 ± 2.1 ^b^	42.7 ± 1.4 ^c^
Length of cannon bone forelimb (LoCBF) [cm]	20.0 ± 1.3 ^a^	21.4 ± 1.9 ^b^	18.6 ± 0.9 ^c^
Length of back (LoB) [cm]	48.3 ± 2.3 ^ab^	49.4 ± 3.6 ^a^	46.1 ± 1.7 ^b^
Height of hock (HoH) [cm]	55.6 ± 3.4 ^ab^	57.3 ± 2.7 ^a^	53.4 ± 1.7 ^b^
Length of cannon bone hindlimb (LoCBH) [cm]	24.1 ± 1.7 ^ab^	25.4 ± 1.6 ^a^	23.5 ± 1.3 ^b^
Circumference of the cannon bone forelimb (CircCBF) * [cm]	19.8 ± 0.6 ^ab^	20.4 ± 0.8 ^a^	19.1 ±1.0 ^b^
Circumference of the cannon bone hindlimb (CircCBH) [cm]	22.1 ± 0.7 ^ab^	22.8 ± 0.8 ^a^	21.5 ± 0.9 ^b^
Difference height at croup—height at withers (Diff_HaC_HaW) [cm]	1.8 ± 2.9 ^ab^	0.2 ± 2.7 ^a^	3.2 ± 3.0 ^b^
Length of withers (LoW) [cm]	26.4 ± 1.7 ^a^	25.9 ± 1.8 ^a^	23.7 ± 2.6 ^b^
Ratio height at withers to circumference of cannon bone hindlimb (Ra_HaW_CircCBH) [cm]	7.13 ± 0.3 ^a^	7.2 ± 0.2 ^a^	6.9 ± 0.3 ^b^
Angle of shoulder (AoS) [°]	96.78 ± 3.0 ^a^	99.5 ± 2.5 ^b^	96.8 ± 3.3 ^a^
Angle of pastern forelimb (AoPF) [°]	63.1 ± 3.7	63.7 ± 3.8	63.2 ± 2.3
Angle of pastern hindlimb (AoPH) [°]	62.2 ± 3.7	63.5 ± 4.6	60.7 ± 3.9
Difference topline—underline (Diff_ToL_UnL) [cm]	1.6 ± 3.5	1.6 ± 3.1	1.7 ± 5.0
Difference hock—carpal joint (Diff_HoH_HoCJ) [cm]	1.2 ± 0.1	1.2 ± 0.1	1.3 ± 0.1
Difference length of cannon bone hindlimb—forelimb (Diff_LoCB) [cm]	4.1 ± 2.0	4.0 ± 2.5	4.9 ± 1.6
Difference angle of pastern forelimb—hindlimb (Diff_AoP) [cm]	0.9 ± 5.0	0.2 ± 5.4	2.5 ± 5.0
Ratio length of cannon bone forelimb to height at withers (Ra_HaW_LoCBF) [cm]	7.9 ± 0.5	7.7± 0.7	8.0 ± 0.4
Ratio length of cannon bone hindlimb to height at withers (Ra_HaW_LoCBH) [cm]	6.6 ± 0.5	6.5 ± 0.4	6.3 ± 0.4
Ratio height at withers to circumference of cannon bone forelimb (Ra_HaW_CircCBF) * [cm]	8.0 ± 0.3	8.1 ± 0.2	7.8 ± 0.3

* Comparison by means using the Kruskal-Wallis Test including post hoc analysis and Bonferroni correction (level of significance *p* < 0.05). Different superscripts in one line demonstrate significant differences.

**Table 4:** Correlations to height at withers of nine variables.

Trait	Correlation Coefficient (*r*)	Significance (2-Sided)
Length of withers (LoW)	0.50	0.000
Length of back (LoB)	0.51	0.000
Difference height at croup minus height at withers (Diff_HaC_HaW)	−0.39	0.008
Length of Cannon Bone forelimb (LoCBF)	0.61	0.000
Length of Cannon Bon hindlimb (LoCBH)	0.47	0.001
Angle of shoulder (AoS)	0.39	0.008
Height of Carpal Joints (HoCJ)	0.79	0.000
Height of hock (HoH)	0.69	0.000
Circumference of the cannon bone hindlimb (CircCBH)	0.66	0.000

## Data Availability

Data sharing not applicable, due to no agreement of the horse owners.

## Supplementary Materials

The following supplementary materials are available online at https://www.mdpi.com/article/10.3390/ani11102861/s1, Figure S1: Experimental setup, Table S1: Crosstable of Chi-Squared Test for HaW in breed description, Table S2: Chi-Squared Test for HaW in breed description, Table S3: Crosstable of Chi Squared Test for HaW compared to HaC. Table S4: Chi Squared Test for HaW compared to HaC.

## References

[1] A. Q. H. Association (2019). Annual Report, Amarillo, Texas. https://www.aqha.com/documents/82601/1589238/2019+AQHA+Annual+Report.pdf/f16217ed-1057-37d1-5138-7927af693d62.

[2] DQHA (2014). Deutsche Quarter Horse Association e.V. https://www.dqha.de/zucht/zuchtziel/.

[3] Meira C.T., Curi R.A., Silva J.A.I.V., Corrêa M.J.M., de Oliveira H.N., da Mota M.D.S. (2013). Morphological and Genomic Differences Between Cutting and Racing Lines of Quarter Horses. J. Equine Vet. Sci..

[4] Avila F., Mickelson J.R., Schaefer R.J., McCue M. (2018). Genome-Wide Signatures of Selection Reveal Genes Associated With Performance in American Quarter Horse Subpopulations. Front. Genet..

[5] Beltrán N.A.R., Meira C.T., De Oliveira H.N., Pereira G.L., Silva J.A.I.V., Da Mota M.D.S., Curi R.A. (2015). Prospection of genomic regions divergently selected in cutting line of Quarter Horses in relation to racing line. Livest. Sci..

[6] Pereira G.L., de Matteis R., Regitano L.C., Chardulo L.A.L., Curi R.A. (2015). MSTN, CKM, and DMRT3 Gene Variants in Different Lines of Quarter Horses. J. Equine Vet. Sci..

[7] Holmström M., Philipsson J. (1993). Relationships between conformation, performance and health in 4-year-old swedish warmblood riding horses. Livest. Prod. Sci..

[8] Barrey E., Desliens F., Poirel D., Biau S., Lemaire S., Rivero J.L.L., Langlois B. (2010). Early evaluation of dressage ability in different breeds. Equine Vet. J..

[9] Ducro B.J., Bovenhuis H., Back W. (2009). Heritability of foot conformation and its relationship to sports performance in a Dutch Warmblood horse population. Equine Vet. J..

[10] Sánchez M.J., Gomez M.D., Peña F., Monterde J.G., Morales J.L., Molina A., Valera M. (2013). Relationship between conformation traits and gait characteristics in Pura Raza Español horses. Arch. Tierzucht.

[11] Jönsson L., Näsholm A., Roepstorff L., Egenvall A., Dalin G., Philipsson J. (2014). Conformation traits and their genetic and phenotypic associations with health status in young Swedish warmblood riding horses. Livest. Sci..

[12] Baban M., Curik I., Antunović B., Cacic M., Korabi N., Mijić P. (2009). Phenotypic Correlations of Stride Traits and Body Measurements in Lipizzaner Stallions and Mares. J. Equine Vet. Sci..

[13] Vicente A., Carolino N., Ralão-Duarte J., Gama L. (2014). Selection for morphology, gaits and functional traits in Lusitano horses: II. Fixed effects, genetic trends and selection in retrospect. Livest. Sci..

[14] Guerrero M.J.S., Molina A., Gomez M.D., Peña F., Valera M. (2016). Relationship between morphology and performance: Signature of mass-selection in Pura Raza Español horse. Livest. Sci..

[15] Schroderus E., Ojala M. (2010). Estimates of genetic parameters for conformation measures and scores in Finnhorse and Standardbred foals. J. Anim. Breed. Genet..

[16] Druml T., Gabdulkhakova A., Artner N., Brem G., Kropatsch W. (2014). The Use of Image Data in the Assessment of Equine Conformation—Limitations and Solutions. Visual Observation and Analysis of Vertebrate and Insect Behavior. https://homepages.inf.ed.ac.uk/rbf/VAIB14PAPERS/druml.pdf.

[17] Gmel A.I., Druml T., Portele K., Von Niederhäusern R., Neuditschko M. (2018). Repeatability, reproducibility and consistency of horse shape data and its association with linearly described conformation traits in Franches-Montagnes stallions. PLoS ONE.

[18] dos Santos M.R., Freiberger G., Bottin F., Chiocca M., Zampar A., Cucco D.D.C. (2017). Evaluation of methodologies for equine biometry. Livest. Sci..

[19] Zechner P., Zohman F., Sölkner J., Bodó I., Habe F., Marti E., Brem G. (2001). Morphological description of the Lipizzan horse population. Livest. Prod. Sci..

[20] Komosa M., Frackowiak H., Purzyc H., Wojnowska A., Gramacki A., Gramacki J. (2013). Differences in exterior conformation between primitive, Half-bred and Thoroughbred horses: Anatomic-breeding approach. Am. Soc. Anim. Sci..

[21] Solé M., Santos R., Gómez M., Galisteo A., Valera M. (2013). Evaluation of conformation against traits associated with dressage ability in unridden Iberian horses at the trot. Res. Vet. Sci..

[22] A. Q. H. Association (2020). Official Handbook of Rules and Regulations.

[23] Galisteo A., Cano M., Morales J., Vivo J., Miró F. (1998). The Influence of Speed and Height at the Withers on the Kinematics of Sound Horses at the Hand-Led Trot. Vet. Res. Commun..

[24] N. R. H. Association (2019). NRHA Handbook.

[25] Brühschwein A., Klever J., Hoffmann A.-S., Huber D., Kaufmann E., Reese S., Meyer-Lindenberg A. (2019). Free DICOM-Viewers for Veterinary Medicine. J. Digit. Imaging.

[26] Kuhnke S., Bär K., Bosch P., Rensing M., von Borstel U.K. (2019). Evaluation of a Novel System for Linear Conformation, Gait, and Personality Trait Scoring and Automatic Ranking of Horses at Breed Shows: A Pilot Study in American Quarter Horses. J. Equine Vet. Sci..

[27] Anderson T.M., McIlwraith C.W. (2004). Longitudinal development of equine conformation from weanling to age 3 years in the Thoroughbred. Equine Vet. J..

[28] Magnusson L.E., Thafvellin B. (1990). Studies on the conformation and related traits of Standardbred trotters in Sweden. J. Anim. Physiol. Anim. Nutr..

[29] Hildebrand M. (1987). The mechanics of horse legs. Am. Sci..

[30] Koenen E., van Veldhuizen A., Brascamp E. (1995). Genetic parameters of linear scored conformation traits and their relation to dressage and show-jumping performance in the Dutch Warmblood Riding Horse population. Livest. Prod. Sci..

[31] Holmstrom M., Magnusson L.-E., Philipsson J. (1990). Variation in conformation of Swedish Warmblood horses and conformational characteristics of élite sport horses. Equine Vet. J..

