# Supplementary material for: Comparison of American Quarter Horses Competing in Western Pleasure, Hunter under Saddle, and Reining Using Linear Traits
Source: Animals (Basel). 2021 Sep 30;11(10):2861. doi: 10.3390/ani11102861 (PMC8532615; doi:10.3390/ani11102861)
Supplement: Supplementary file 1 [file animals-11-02861-s001.zip › animals-1292516-supplementary.pdf]

animals

Supplementary Materials: Comparison of American Quarter Horses Competing in Western Pleasure, Hunter under Saddle, and Reining Using Linear Traits

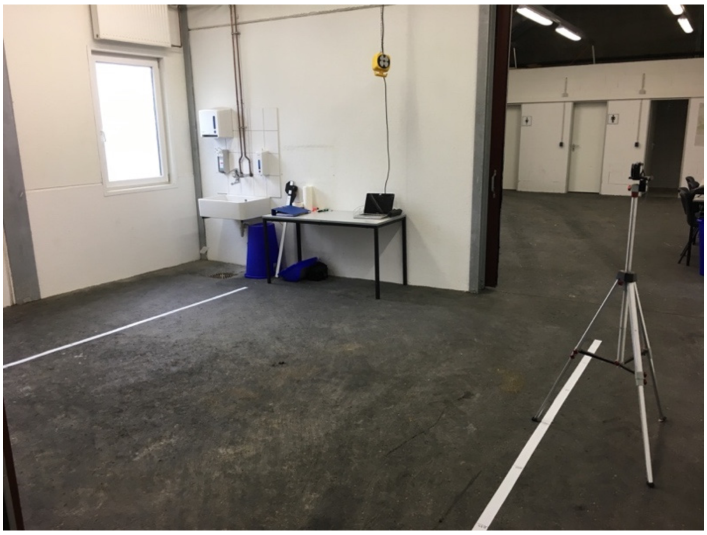

Figure S1: Experimental setup

Table S1Crosstable of Chi-Squared test for HaW in breed description

Crosstable				
		HaW outside breed description		
		No	Yes	Total
Group	PL	15	0	15
	HUS	8	7	15
	RN	14	1	15
Total		37	8	45

Table S2: Chi-Squared Test for HaW in breed description

Chi-squared-tests			
	value	df	asymptotic significance (two-sided)
Chi-squared after Pearson	13.074[a]	2	.001
Likelihood-quotient	14.045	2	.001
connection linear with linear	.233	1	.637
Number of valid cases	45		

a. 3 cells (50.0%) have an expected frequency less than 5. the minimum expected frequency is 2.67.

Table S3: Crosstable of Chi Squared Test for HaW compared to HaC.

**Crosstable**				
		HaW outside breed description		
		No	Yes	Total
Group	PL	5	10	15
	HUS	8	7	15
	RN	3	12	15
Total		16	29	45

Table S4: Chi Squared Test for HaW compared to HaC

Chi-squared-tests			
	value	df	asymptotic significance (two-sided)
Chi-squared after Pearson	3.685	2	.158
Likelihood-quotient	3.738	2	.154
connection linear with linear	.569	1	.451
Number of valid cases	45		

a. 3 cells (50.0%) have an expected frequency less than 5. the minimum expected frequency is 5.33.
